# Bidirectional Long Short-Term Memory–Based Detection of Adverse Drug Reaction Posts Using Korean Social Networking Services Data: Deep Learning Approaches

**DOI:** 10.2196/45289

**Published:** 2024-11-20

**Authors:** Chung-Chun Lee, Seunghee Lee, Mi-Hwa Song, Jong-Yeup Kim, Suehyun Lee

**Affiliations:** 1 Department of Biomedical Informatics, College of Medicine, Konyang University, Daejeon, Republic of Korea; 2 Healthcare Data Science Center, Konyang University Hospital, Daejeon, Republic of Korea; 3 Division of Computer Engineering, College of IT Engineering, Hansung University, Seoul, Republic of Korea; 4 Department of Computer Engineering, College of IT Convergence, Gachon University, Seongnam, Republic of Korea

**Keywords:** adverse drug reaction, social network service, classification model, Korean text data, social networking service, drug detection, deep learning, Korea, social data, older, older adults, drug surveillance, medicine

## Abstract

**Background:**

Social networking services (SNS) closely reflect the lives of individuals in modern society and generate large amounts of data. Previous studies have extracted drug information using relevant SNS data. In particular, it is important to detect adverse drug reactions (ADRs) early using drug surveillance systems. To this end, various deep learning methods have been used to analyze data in multiple languages in addition to English.

**Objective:**

A cautionary drug that can cause ADRs in older patients was selected, and Korean SNS data containing this drug information were collected. Based on this information, we aimed to develop a deep learning model that classifies drug ADR posts based on a recurrent neural network.

**Methods:**

In previous studies, ketoprofen, which has a high prescription frequency and, thus, was referred to the most in posts secured from SNS data, was selected as the target drug. Blog posts, café posts, and NAVER Q&A posts from 2005 to 2020 were collected from NAVER, a portal site containing drug-related information, and natural language processing techniques were applied to analyze data written in Korean. Posts containing highly relevant drug names and ADR word pairs were filtered through association analysis, and training data were generated through manual labeling tasks. Using the training data, an embedded layer of word2vec was formed, and a Bidirectional Long Short-Term Memory (Bi-LSTM) classification model was generated. Then, we evaluated the area under the curve with other machine learning models. In addition, the entire process was further verified using the nonsteroidal anti-inflammatory drug aceclofenac.

**Results:**

Among the nonsteroidal anti-inflammatory drugs, Korean SNS posts containing information on ketoprofen and aceclofenac were secured, and the generic name lexicon, ADR lexicon, and Korean stop word lexicon were generated. In addition, to improve the accuracy of the classification model, an embedding layer was created considering the association between the drug name and the ADR word. In the ADR post classification test, ketoprofen and aceclofenac achieved 85% and 80% accuracy, respectively.

**Conclusions:**

Here, we propose a process for developing a model for classifying ADR posts using SNS data. After analyzing drug name-ADR patterns, we filtered high-quality data by extracting posts, including known ADR words based on the analysis. Based on these data, we developed a model that classifies ADR posts. This confirmed that a model that can leverage social data to monitor ADRs automatically is feasible.

## Introduction

The frequency and quantity of drugs taken worldwide due to aging are rapidly increasing. As a result, adverse drug reactions (ADRs) have also increased rapidly, threatening the safety of patients. Accordingly, the identification and early detection of new or serious ADR in drugs on the market is an increasingly important issue [1].

Data from social networking services (SNS) is significant because it contains information related to, or indicating, known ADRs, in addition to unknown ADRs [2]. In Europe, the Innovative Medicines Initiative World Wide Web-Recognizing Adverse Drug Reactions (WEB-RADR) project was carried out during 2014‐2017 to address questions about the potential use of social media to monitor ADRs. Whether social media is as valuable as other data sources, such as voluntary reporting, in ADR monitoring has been studied [3].

Various analyses have been attempted by collecting text referring to ADRs, drugs, or health conditions from SNS data. These studies have played a large part in defining and mapping terminology to standard terms for drugs, symptoms, and ADRs, and as a result of this, studies have recently been conducted to detect ADRs using deep learning [4]. There has also been a study on classifying ADR posts through architectures such as Hierarchical Attention Networks, FastText, and Convolutional Neural Network–based on word2vec in a dataset consisting of 11,623 posts related to drugs on Ask a Patient and Twitter [5]. To improve the performance of the model for classifying ADR posts, there are concerns regarding the data balance and feature selection, which must be overcome. To address this problem, they adopted Bidirectional Encoder Presentations from Transformers (BERT) to introduce an architecture that selects keywords, such as those entered as layers before the classification model, and was recognized by humans [6].

In addition, studies have focused on mapping consumer and medical terms of posts collected from social channels [7]. Creating an ADR post classification model based on medical terminology from databases such as Systematized Nomenclature of Medicine Clinical Terms (SNOMED CT) or The Medical Dictionary for Regulatory Activities (MedDRA) creates a clinical foundation for future applications and can be used for various drugs and ADRs.

Using Chinese social media posts as the original dataset, we proposed a semisupervised learning framework for detecting Chinese drug terms and ADR terms [8]. Russian Drug Reaction Corpus (RuDReC) is a Russian corpus labeled with drug terms, disease information, health terms, drug efficacy, drug adaptation, drug ADRs, and more. In addition, an experiment to automate object recognition in this corpus achieved an *F*_1_-score of approximately 74.85% [9].

Herein, we introduce a process consisting of collecting Korean posts containing drug information on social channels, selecting posts containing essential information among the collected posts, and creating and evaluating a model for classifying ADR posts. Therefore, the final purpose of this study was to create a model that effectively classifies ADR posts, written in Korean, for drugs with a high frequency of use among senile individuals.

This requires natural language preprocessing in Korean and is verified, not only for target drugs but also for additional drugs in the process of generating a classification model for specific ADR posts.

## Methods

### Overview

**Figure 1.**
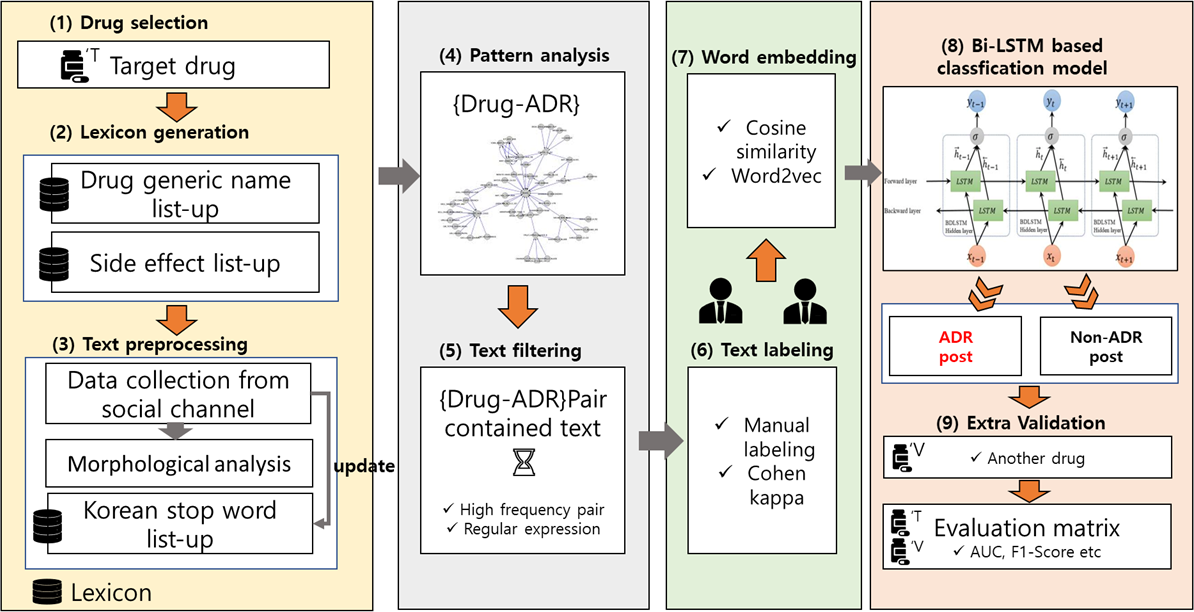
Overview of this research. ADR: adverse drug reaction; AUC: area under the curve; Bi-LSTM: Bidirectional Long Short-Term Memory.

In this study, we propose a pipeline for classifying drug ADR posts using a circular neural network-based classification model based on SNS data. In summary, first, a social channel suitable for drug monitoring was selected. Second, the generic name of the target drug, ADR lexicon, was generated. Third, text data were obtained from social channels based on the generic name lexicon for the drug for analysis. Morphological analysis was conducted using the obtained text data, and unnecessary Korean terms were added to the stop word lexicon, simultaneously. Fourth, the pattern of drug ADRs was analyzed by association analysis and embedding analysis of text data that had undergone preprocessing and term filtering. Fifth, posts containing ADR words identified by analysis of drug ADR patterns were filtered. Sixth, 2 researchers manually labeled the collected text data and verified their reliability. Seventh, we created an embedding layer that considered the correlation between generic names and ADR words. Eighth, we developed a recurrent neural network (RNN)–based classification model using a labeled dataset. Finally, to verify the generated classification model, additional drugs were selected, and the entire step was repeated. Subsequently, the model was evaluated using a matrix that evaluated the target drug and additional drugs. Figure 1 shows the whole workflow of this study.

### Dataset

A previous study [10] calculated the Beers Criteria drug prescription and the incidence of side effects in persons aged 65 years or older in Korea. The top 3 drugs (metoclopramide, chlorpheniramine, and ketoprofen) were selected based on prescriptions and side effects reported in the clinical environment of Konyang University Hospital [11]. Therefore, the focus of this study was on ketoprofen.

### Lexicon Generation

#### Generic Name Lexicon

We searched for ketoprofen based on the information provided by the Korea Pharmaceutical Information Service (KPIS). After searching for the target drug, the drug name lexicon was generated by collecting the searched drug brand names.

#### ADR Lexicon

The ADR lexicon was generated using a list of drug ADRs based on a standardized and published database of drug ADR, WHO-ART [12], SIDER [13], and a lexicon of pregenerated consumer terms. The WHO-ART represents an international classification system for drug ADR terms and is the most widely used system in ADR reporting.

Accordingly, the Korean version of WHO-ART version 092 has been produced by the Korea Institute of Drug Safety and Risk Management (KIDSRM) [14]. SIDER is a drug ADR database that includes information on drug-ADR relationships and drug ADRs to commercially available drugs. After searching for “ketoprofen” in SIDER, an ADR lexicon was established by mapping definitions from the listed MedDRA preferred term and WHO-ART version 092 Korean version.

### Text Preprocessing

#### Data Collection

When securing data using the Naver Open API (NAVER Developers), there is a limit to the number of posts that can be secured. To address this, page information from search results on café posts, blogs, and Naver Q&A platforms was used. Only the body content of each post was collected from its URL on the page, while sensitive information, such as user IDs and café names, was excluded.

#### Morphological Analysis

**Figure 2.**
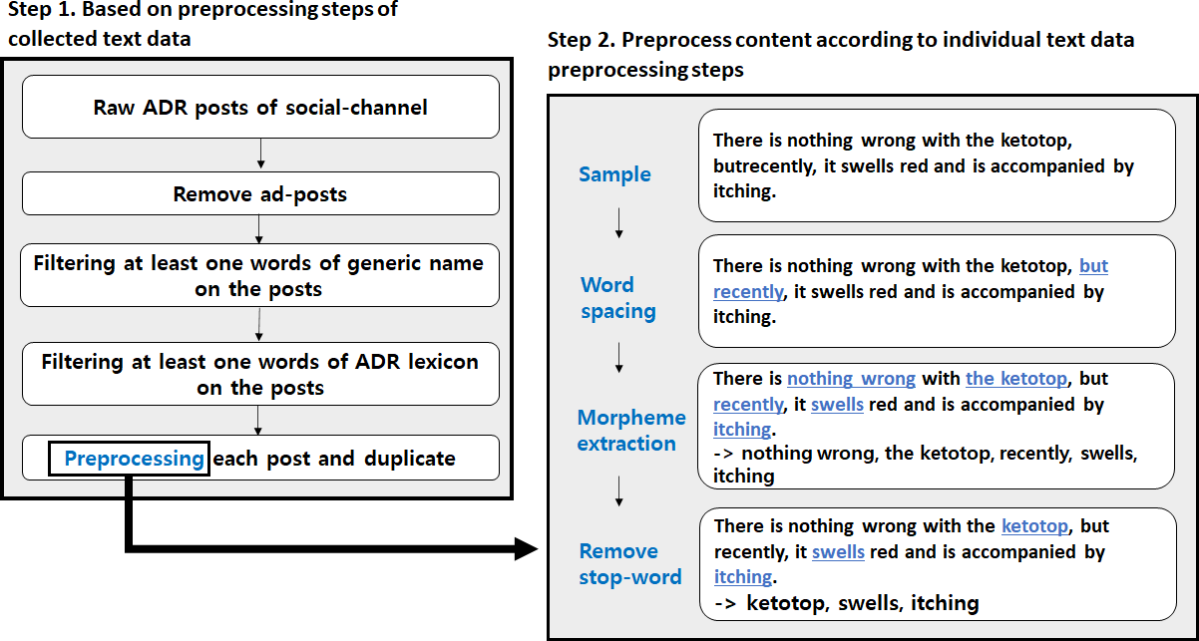
Detailed steps of text preprocessing. ADR: adverse drug reaction.

In the case of SNS posts, it is common to find cases in which spaces between words are not included, or morphemes are excluded for quick communication and convenience. These errors can adversely affect the analysis results; therefore, preprocessing is necessary. The SoySpacing module was used for the spacing preprocessing, and additional spacing rules were applied and updated to the module to reduce the derived errors. The morpheme analyzer performed word tokenization using the Korean Intelligent Word Identifier module [15]. Words extracted through tokenization may contain many meaningless nouns that do not meet the purpose of the study; these words were selected and updated in the stop-word lexicon. The detailed process of text preprocessing using the collected data is shown in Figure 2.

### Pattern Analysis

#### Association Analysis

Association analysis finds interrelationships or dependencies within data, enabling a simple and clear interpretation of results [16]. The System-Organ Class (SOC) ranking of 960 patients (2013‐2017) who reported spontaneous ADRs with ketoprofen from the Korea Adverse Event Reporting System (KAERS) database confirmed that the adverse reactions found in the SNS-based pattern analysis had similar patterns to the upper SOC group.

#### Filtering ADR Posts

Labeled text datasets are required to develop an ADR posting classification model. After preprocessing the collected data, we filtered the posts containing the generic drug words and drug ADR pairs that were significant in the pattern analysis.

### Training Dataset

#### Labeling ADR Posts

Two researchers majoring in information medicine were recruited to manually label the filtered posts. In the case of drug ADR posts, 1 nondrug ADR post was classified as 0, and the reliability of workers was evaluated using kappa analysis (Cohen or Pleiss kappa Coordinator).

#### Embedding Layer Created From Filtered Posts

We created an embedding model using an embedding technique (word2vec) for posts filtered through association analysis. The embedding model includes the association between the ADR terms “Ketotop” and “Antipuramin.” The embedding model was applied as the input layer of the Bi-LSTM model.

### Development Classification Model

#### RNN-Based Classification Model

By applying deep learning techniques to SNS posts, the classification model creation process was established by referring to existing studies [4] to detect drug ADR posts. In the same context as time-series data with an order of events, text data are composed of a sequence for each word in a sentence. To this end, we used the RNN model, which uses the Long Short-Term Memory (LSTM) model to create a model that classifies whether drug ADR postings are recent [17]. Bi-LSTM considers the sequence of input words in both directions by adding an additional Hidden State (backward direction) to the LSTM’s Hidden State (forward direction). The system used Python (version 3.8; Python Software Foundation) and Tensorflow (Google Brain Team), and the PC environment consisted of an Intel Core i7-8700 3.2 GHz CPU and 16 GB RAM.

The machine-learning procedure of the Bi-LSTM model is summarized as follows. First, preprocessing is performed on the labeled text data, where words are tokenized for each post through morpheme analysis. Second, frequency-based integer encoding of the corresponding text data and padding steps for parallel processing were performed. Third, the input text data were embedded to fit the embedding length in the embedding layer. Fourth, the number of hidden units was set to 256. Finally, the RNN model solving the classification problem uses multi-to-one, for which the output of the model was constructed with binary classification, the activation function was set to sigmoid, the optimizer was set to Adam, and the additional parameters were learning rate=0.001, batch_szie=64, and epoch=50.

**Figure 3.**
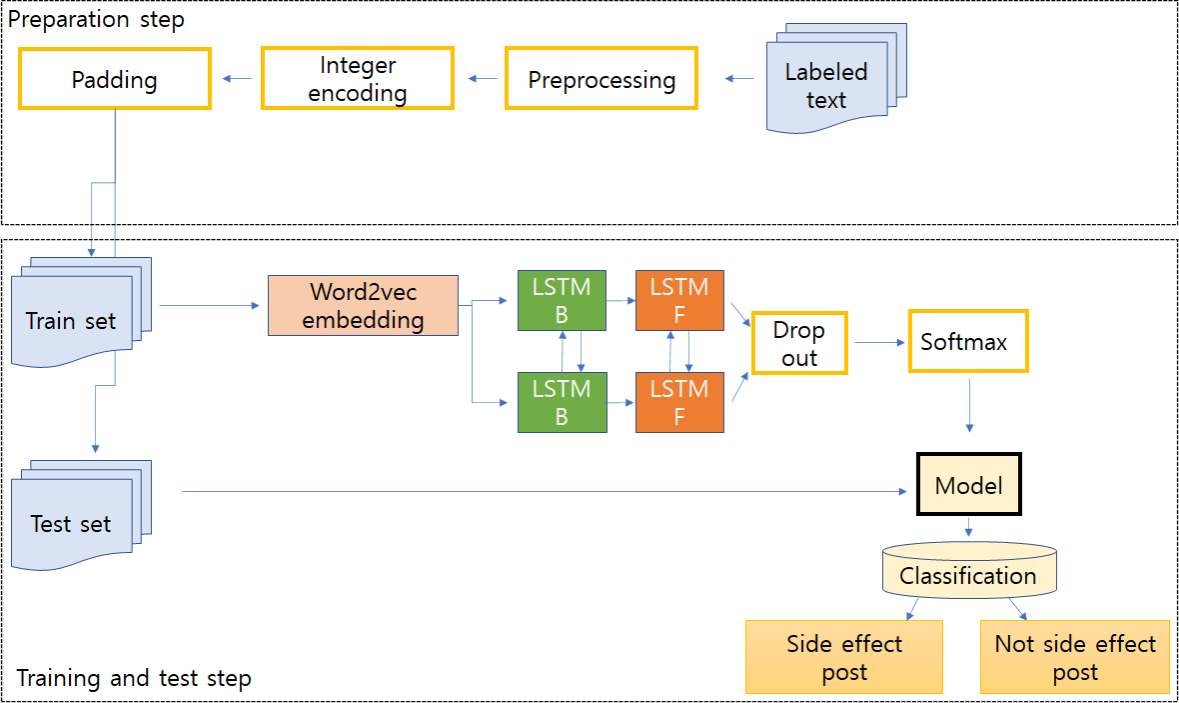
The framework of the Bi-LSTM–based classification approach. Bi-LSTM: Bidirectional Long Short-Term Memory; LSTM: Long Short-Term Memory.

The training data and test data were divided by a ratio of 7:3, and the output value classification criterion (threshold) in the binary classification prediction was a default of 0.5. The procedure of deep learning model development is presented in Figure 3.

#### Extra Drug Validation

The generation of the classification model is the last step of the process, and the entire step verified an extra drug. Based on the Korea Ministry of Food and Drug Safety’s press release, “What medicines should senior citizens be careful of?” [18], the extra drug selected was a nonsteroidal anti-inflammatory drug, as is ketoprofen. Among the nonsteroidal anti-inflammatory drugs reported in the press release, a similar amount of data to that of ketoprofen was secured for Geworin (generic name). Therefore, Geworin was determined as the extra drug.

#### Comparison of the Other Classification Method

As a validation method for Bi-LSTM machine learning techniques, in our study, classification models were generated and executed on the same dataset using Gated Recurrent Units, Convolutional Neural Network-Bidirectional Long Short-Term Memory, and Korean Generative Pre-trained Transformer 2.

### Ethical Considerations

The data used in our study are publicly available and fully anonymized, and no personal or identifiable information has been collected or used during the research process. Given the nature of the data and the absence of any human participant involvement or personal data processing, this research does not fall under the scope of studies requiring institutional review board approval, according to relevant ethical guidelines.

## Results

### Data Acquired

From 2005 to 2020, a total of 11,693 posts were collected through crawling collectors. The ketoprofen posts that underwent the pretreatment process are described in “Text Preprocessing.” These were used for pattern analysis, resulting in the final 3828 posts. Table 1 shows 1 example.

**Table 1.** Example of brand name and ADR^a^ of ketoprofen posts.

Class	Context
Drug	Last year and the year before last, I used Antiphlamine_6_drug for insect bites and muscle pain. Strangely, I don’t think I can feel as strong a battle as before.
ADR	When I got home, I got blisters_153_ADR and it was crazy. Also itch_156_ADR I’m going to go to the hospital.

a ADR: adverse drug reaction.

### Generated Lexicon

For ketoprofen, 3 types of lexicons (generic name, ADR, and stop word) were generated. The drug name lexicon includes 9 drug names such as Ketotop and Antiphlamine, and the ADR lexicon includes 2925 ADR terms such as “rash,” “swelling,” and “stomach.” Finally, the stop word lexicon consists of 7196 words such as “opposite,” “everyone,” and “more.”

### ADR Pattern Analysis Results

Pattern analysis was performed using R (version 4.1.0; R Core Team). We examined the correlation between ketoprofen and its ADR terms based on the association analysis and found the highest correlation (support:0.01, confidence:0.6) was with “muscle pain” and “disability,” while correlation analysis of Antiphlamine showed the highest correlation with “dryness” and “allergy.” Although these support values are low, this study analyzed data with a relatively low frequency, which also has an impact.

### Embedding Model of Filtered Post

When creating an embedding model for filtered posts, the parameters used in word2vec were skip-gram and 300 dimensions. Through this, it was found that there was a high degree of cosine similarity between “ketotop” and “itching,” and “heat rash” and “rash.” In addition, antipuramin had a high degree of cosine similarity with “itching,” “burning,” and “swelling.”

### Model Classification Results

Two researchers majoring in information medicine manually labeled the text data for training data generation. The labeled text consisted of 576 posts containing a significant drug name and drug ADR word pair after pattern analysis, indicating reliability with a kappa score of 0.8. Table 2 contains all post numbers of ketoprofen's final dataset. The test accuracy of the model was 85%. Table 3 presents the results of the model tests.

**Table 2.** Manually labeled text table.

Category		Ketoprofen		Total (N=576)
		Antipuramin (n=403), n (%)	Ketotop (n=173), n (%)	
**Labeling**				
	Non-ADR^a^ post	229 (56.8)	103 (59.5)	332 (57.6)
	ADR post	174 (43.2)	70 (40.5)	244 (42.4)

a ADR: adverse drug reaction.

**Table 3.** Confusion matrix.

Category		Prediction, n		Total, n
		Non-ADR^a^ post	ADR post	
**Actual**				
	Non-ADR post	93	8	101
	ADR post	18	54	72

a ADR: adverse drug reaction.

The results showing the receiver operating characteristic (ROC) curve of the model for each binary classification threshold point are as follows. In terms of accuracy, the highest accuracy was approximately 85% when the threshold point was 0.5, and the lowest accuracy was approximately 58% when the threshold point was 1.0. The threshold point in the table above was 0.5, and the accuracy was 85%. In the case of recall, 54 out of 72 ADR posts were matched to ADR posts to achieve an accurate prediction of 75%.

### Extra Drug Validation

For the collected data of the additional drug, Geworin, 66 (27.96%) posts referred to ADRs and 170 (72%) posts referred to non-ADRs out of the total of 236 posts. The results of the experiment with the same Bi-LSTM architecture after dividing the training and test data by 7:3. The accuracy of this test was 80%. Table 4 shows the classification result as a confusion matrix of Geworin test data.

**Table 4.** Extra drug confusion matrix.

Category		Prediction, n		Total, n
		Non-ADR^a^ post	ADR post	
**Actual**				
	Non-ADR post	42	5	47
	ADR post	9	15	24

a ADR: adverse drug reaction.

**Figure 4.**
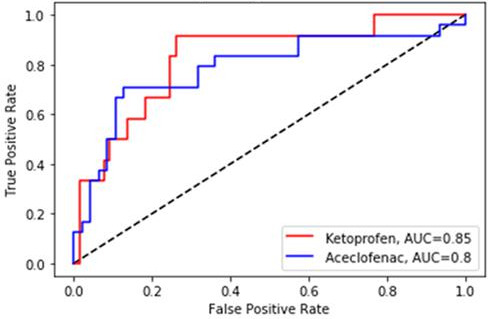
Receiver operating characteristic analysis of 2 drugs to identify posts on adverse drug reaction. AUC: area under the curve.

The results showing the ROC curve of the model for each binary classification threshold point are as follows (see Figure 4). The largest accuracy was about 82% when the threshold point was 0.3, and the lowest accuracy was about 60.5% when the threshold point was 0.1. The threshold point in the table above was 0.5, and the accuracy was 80%. In the case of recall, 15 of the total 24 ADR posts were matched to make an accurate prediction of about 62% and decreased compared to the results of ketoprofen-containing drugs (75% to 62%).

### Comparison for the Other Classification Method

**Figure 5.**
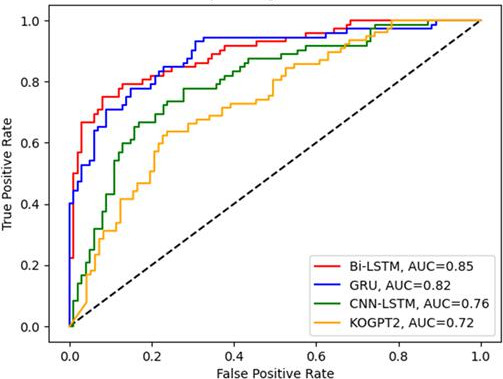
Receiver operating characteristic analysis of Bi-LSTM with other classification models. AUC: area under the curve; Bi-LSTM: Bidirectional Long Short-Term Memory; CNN: Convolutional Neural Network; GRU: Gated Recurrent Units; KOGPT2: Korean Generative Pre-trained Transformer 2; LSTM: Long Short-Term Memory.

In a classification of ADR posts, other classification model methods other than Bi-LSTM used in our study were applied and performance indicators were compared among deep learning. It was found that Bi-LSTM showed good performance overall. In particular, Korean Generative Pre-trained Transformer 2 was a transformer-based classification model and was a specialized method for Korean, but showed the lowest performance. The models’s parameters were learning rate=0.001, batch_szie=64, and epoch=50. Also, training data and test data were divided by a ratio of 7:3. It is the same with Bi-LSTM. We experimented using Python’s early stopping module. This led to the derivation of the optimal area under the curve value. The comparison of ROC curve is shown in Figure 5.

Table 5 shows the classification result including other classification method.

**Table 5.** Comparison of Bi-LSTM^a^ with other classification models.

Antipuramin and ketotop	Bi-LSTM	GRU^b^	CNN-Bi-LSTM^c^	KOGPT2^d^
Precision	0.87	0.79	0.73	0.69
Recall	0.75	0.76	0.67	0.66
F1-score	0.81	0.77	0.70	0.68
AUC^e^	0.85	0.82	0.76	0.72

a Bi-LSTM: Bidirectional Long Short-Term Memory.

b GRU: Gated Recurrent Units.

c CNN: Convolutional Neural Network.

d KOGPT2: Korean Generative Pre-trained Transformer 2.

e AUC: area under the curve.

## Discussion

### Principal Findings

The model proposed in this study uses a Bi-LSTM–based classification model to extract drug ADR posts from SNS data and evaluate their significance. We investigated whether these processes can be effectively used for drug monitoring and proposed a classification model development process for extracting Korean ADR posts based on stepwise methods. We comprehensively reviewed the advantages and disadvantages of using social media data for drug ADR detection [19] and investigated whether social media analysis can be integrated with voluntary reporting systems to improve ADR detection [20]. No previous studies have performed drug monitoring using KAERS, a Korean voluntary reporting system, or NAVER, a social channel. Social media conversations also provide a wide range of information about the patient’s health, such as drug use information. This may explain diversity in the data in terms of ADRs other than the known ADR of the drug [21]. When using NAVER posts, data were collected from 2 channels (Naver Blogs and Naver Café, Naver Q&A platform) to obtain the actual voices of consumers, not studies that contain general information. First, for famous drug names, SNS data can be used to obtain sufficient ADR data, suggesting that they can be used in drug ADR studies or drug surveillance studies. Second, we proposed a method to build a Korean ADR lexicon based on the mapping of the SIDER DB results with WHO-ART files (including Korean terms) for target drugs. Third, it was confirmed that the pattern of drug ADRs obtained using SNSs was not different from the SOC range of self-reported ADR information obtained from KAERS. This finding adds credibility to our method. Fourth, based on the results of pattern analysis, we present the process of filtering posts and generating them as artificial intelligence learning data, and propose a model that uses them to classify posts regarding ADRs of target drugs. Despite older people having reduced use of internet-based platforms, this may not limit the data as information shared with family members may appear on social networks. However, this study has several other limitations. First, data were only collected from Naver posts; therefore, information was limited to those users, and other platforms such as Instagram (Meta Platforms) and Twitter (Twitter, Inc) were not considered. Second, only the known ADRs of the target drug were defined when constructing the lexicon. An extension of the unknown ADR lexicon is required to extract unknown ADRs to aid drug monitoring systems. Third, there is insufficient evidence to prove that filtered and labeled posts are clinically associated with drug ADRs. This requires a review by clinical experts during the labeling of posts. Fourth, the depth of the classification model development phase is only at the initial experimental level. The researchers proposed a model to classify posts referring to ketoprofen-related ADRs on social media using well-known ADR information that can be further studied, including unknown ADRs. Furthermore, the model will be upgraded in the future by applying various techniques to resolve false negativity. The target drugs analyzed will also be expanded to high-demand drugs, such as tylenol and aspirin. The overall process, from the proposed collection to model development, can detect ADRs from a consumer’s perspective from outside the hospital, which can be used to inform drug safety monitoring policies for a specific drug.

### Conclusions

In this study, we proposed a process for developing a model for classifying drug ADR posts using SNS data. After analyzing drug ADR patterns, we filtered high-quality data by filtering posts including known drug ADR terms based on the results. Based on these data, we developed a model that classifies drug ADR posts. This confirmed that a model that can leverage social media data to automatically monitor drug ADRs is feasible.

## Abbreviations

ADR: adverse drug reaction
BERT: Bidirectional Encoder Presentations from Transformers
Bi-LSTM: Bidirectional Long Short-Term Memory
KAERS: Korea Adverse Event Reporting System
KIDSRM: Korea Institute of Drug Safety and Risk Management
KPIS: Korea Pharmaceutical Information Service
LSTM: Long Short-Term Memory
MedDRA: Medical Dictionary for Regulatory Activities
RNN: recurrent neural network
ROC: receiver operating characteristic
RuDReC: Russian Drug Reaction Corpus
SNOMED CT: Systematized Nomenclature of Medicine Clinical Terms
SNS: social networking service
WEB-RADR: World Wide Web-Recognizing Adverse Drug Reactions
